# pH/Thermo-Responsive Grafted Alginate-Based SiO_2_ Hybrid Nanocarrier/Hydrogel Drug Delivery Systems

**DOI:** 10.3390/polym13081228

**Published:** 2021-04-10

**Authors:** Nikolaos Theodorakis, Sofia-Falia Saravanou, Nikoleta-Paraskevi Kouli, Zacharoula Iatridi, Constantinos Tsitsilianis

**Affiliations:** Department of Chemical Engineering, University of Patras, 26500 Patras, Greece; nikolastheo96@hotmail.com (N.T.); faliasaravanou@hotmail.com (S.-F.S.); nikoleta.li.paraleta@gmail.com (N.-P.K.)

**Keywords:** thermo-responsive graft copolymer, alginate, PNIPAM, thermo-thickening, shear thinning injectability, organic/inorganic hybrid, mesoporous silica, LbL technique, hydrogel composite

## Abstract

We report the preparation of mesoporous silica nanoparticles covered by layer by layer (LbL) oppositely charged weak polyelectrolytes, comprising poly(allylamine hydrochloride) (PAH) and a sodium alginate, highly grafted by *N*-isopropylacrylamide/*N*-tert-butylacrylamide random copolymers, NaALG-g-P(NIPAM_90_*-co-*NtBAM_10_) (NaALG-g). Thanks to the pH dependence of the degree of ionization of the polyelectrolytes and the LCST-type thermosensitivity of the grafting chains of the NaALG-g, the as-prepared hybrid nanoparticles (hNP) exhibit pH/thermo-responsive drug delivery capabilities. The release kinetics of rhodamine B (RB, model drug) can be controlled by the number of PAH/NaALG-g bilayers and more importantly by the environmental conditions, namely, pH and temperature. As observed, the increase of pH and/or temperature accelerates the RB release under sink conditions. The same NaALG-g was used as gelator to fabricate a hNP@NaALG-g hydrogel composite. This formulation forms a viscous solution at room temperature, and it is transformed to a self-assembling hydrogel (sol-gel transition) upon heating at physiological temperature provided that its T_gel_ was regulated at 30.7 °C, by the NtBAM hydrophobic monomer incorporation in the side chains. It exhibits excellent injectability thanks to its combined thermo- and shear-responsiveness. The hNP@NaALG-g hydrogel composite, encapsulating hNP covered with one bilayer, exhibited pH-responsive sustainable drug delivery. The presented highly tunable drug delivery system (DDS) (hNP and/or composite hydrogel) might be useful for biomedical potential applications.

## 1. Introduction

Mesoporous silica nanoparticles (MSNs) have been broadly used as biomaterials owing to their advantageous characteristics like good biocompatibility, large surface area, well-defined mesoporous structure with tunable pore size, high drug encapsulation efficiency, and reactive surface [1,2,3,4,5,6,7,8,9,10]. However, a major drawback of the MSNs is the inability to control the release of the encapsulated payload. In order to overcome this obstacle, surface functionalization of the silica particles is a prerequisite. The modification of the silica surface with polymers is a key method that offers improved and additional properties to the silica particles [11,12,13,14].

While many synthetic polymers have been used all over the years to modify the MSNs surface, in recent years, there has been a trend in the utilization of natural polymers in order to combine the polymeric nature as well as the features of natural polymers like for instance biocompatibility. Many paradigms report the fabrication of hybrid composites comprising the inorganic MSNs and the organic natural polymer sodium alginate (NaALG). NaALG is an extensively studied natural polysaccharide because of a plethora of advantages it exhibits like biocompatibility, low toxicity, biodegradability, ease to functionalize and good gelation properties [15,16,17,18]. NaALG has found use in many fields like biomedical applications and therapeutics (drug encapsulation and delivery, tissue regeneration), in the food industry or in environmental applications [19,20,21,22,23,24]. Due to the numerous free carboxyl groups along its backbone, it acts as an anionic polyelectrolyte with pH-responsive properties. Moreover, these carboxyl groups can easily be modified in order to produce graft copolymers or other alginate derivatives with improved characteristics [25,26].

Alginate can be covalently or non-covalently bonded on the MSNs surface. In the first case, methods like grafting onto the silica particles, polymerization in the presence of silica particles or crosslinking are followed. Some examples of covalently attached ALG on silica particles are listed below. Prochloraz functionalized silica microcapsules were prepared by cross-linking silica with ALG using carbodiimide chemistry [27]. The surface of anisotropic silica was grafted with ALG through a catalyst-free Ugi reaction [28]. In another work, NaALG was used to prepare end-capped MSNs by disulfide bonds, for the fabrication of stimuli-responsive nanocarrier for drug delivery [29]; de Lima et al. prepared a vinyl-functionalized ALG that could crosslink with a vinyl-functionalized mesoporous silica. This led to the formation of a hybrid network consisting of a polysaccharide hydrogel that contains mesoporous silica, for enhanced drug delivery applications [30].

In the second scenario, the MSNs surface is functionalized through non-covalent associations (like ionic bonds, hydrogen bonds, etc). Hybrid microspheres comprising inorganic MSNs and organic ALG (MSN@Alg) reported by Liao et al. exhibited effective biocompatibility and good therapeutic agent (anti-cancer drug, organic dye) loading capacity and sustained release. The functionalization with cancer cell targeting peptides rendered them excellent intracellular delivery efficacy [31]. Feng et al. reported the construction of a pH-responsive anti-tumor doxorubicin (DOX) drug delivery system by modifying MSNs surface with two polyelectrolyte multilayers of NaALG and chitosan [32]. The produced MSNs had enriched efficacy and biocompatibility. In another work, carboxyl modified MSNs were coated with a modified chitosan (modification with the fluorescent molecule fluorescein isothiocyanate (FITC)) and NaALG through LBL. Afterwards, the LBL covered MSNs were PEGylated, and the final hybrid nanoparticles could efficiently encapsulate DOX and showed pH-responsive controlled release of DOX. Moreover, the presence of FITC rendered the hybrid nanoparticles cell imaging capabilities and potential use in applications like cancer treatment [33]. More recently, Au, Fe_3_O_4_ nanoparticles, DOX, the photosensitizer chlorin e6 (Ce6) and small hairpin RNA (P-gp shRNA) were all combined into MSNs coated with polyelectrolyte layers of NaALG and chitosan by the LBL self-assembly technique, offering a multifunctional delivery nanocarrier with imaging, diagnosis and therapy capabilities [34].

In a previous study, we synthesized an alginate-based comb-type gelator with thermo- and shear-induced responsive capabilities [35]. This sodium alginate is grafted by a thermo-responsive, LCST-type (Lower Critical Solution Temperature) amphiphilic copolymer of *N*-isopropylacrylamide with *N*-tert-butylacrylamide (P(NIPAM_90_-*co*-NtBAM_10_)-NH_2_), NaALG-*g*-P(NIPAM_90_-*co*- NtBAM_10_). In aqueous media the graft copolymer self-assembles forming a physically crosslinked 3D network, which is due to the thermo-induced hydrophobic association of its P(NIPAM_90_*-co-*NtBAM_10_) side chains, that form the physical crosslinks. The PNIPAM enrichment with the 10 mol% with the hydrophobic NtBAM provides some advantages, namely, regulation of the LCST [36] and in turn the gelation temperature and strengthening of the hydrophobic interactions responsible for the formation of the network crosslinks. The synergy of both thermo- and shear-responsiveness rendered this hydrogel with promising injectable self-assembling capability [37] that has great potential to be used in bioapplications.

The present work concerns the design and fabrication of MSN-based nanocarriers layered, through the LbL technique, by oppositely charged polyelectrolytes of poly(allylamine hydrochloride (PAH) as the cationic and NaALG-*g*-P(NIPAM_90_-*co*-NtBAM_10_) (NaALG-g) graft copolymer with 86 grafting chains per alginate backbone, as the anionic polymer constituents. The innovation of this design arises from the incorporation of the P(NIPAM_90_-*co*-NtBAM_10_) side chains in the anionic polyelectrolyte layer, endowing the nanocarrier with thermo-responsiveness [36]. The as-prepared nanoparticulate drug delivery system (DDS) was evaluated in vitro using the fluorescence rhodamine B (RB) as model hydrophilic drug. The delivery of RB can be controlled by the number of bilayers (PAH/NaALG-g), and the environmental conditions, namely, temperature and pH. Moreover, the MSN@PAH/NaALG-g nanoparticles, loaded with RB, were encapsulated within a NaALG-*g*-P(NIPAM_90_-*co*-NtBAM_10_) injectable hydrogel, fabricating a hydrogel composite DDS. This system exhibits sustainable RB release, controlled again by temperature and pH. Importantly, the temperature-controlled release of RB is now governed by the thermo-responsive hydrogel properties, which differs from those of the neat hybrid nanoparticles.

## 2. Materials and Methods

### 2.1. Materials

The monomers *N*-isopropylacrylamide (NIPAM, Fluorochem, Derbyshire, UK) and *N*-tert-butylacrylamide (NtBAM, Alfa Aesar, Ward Hill, MA, USA) were used as received. The polymers sodium alginate (NaALG, Sigma-Aldrich, St. Louis, MO, USA, No. 180947, molecular weight range: 120,000–190,000 g/moL and mannuronic/guluronic ratio (M/G): 1.53 (values given by provider)) and poly(allylamine hydrochloride) (PAH) were bought from Sigma-Aldrich (St. Louis, MO, USA). NaALG was purified and characterized by intrinsic viscosity method to determine the viscosity average of the molecular weight, Mv (the purification procedure is the same as described in a previous work [35]). 2-Mercaptoethylamine hydrochloride, potassium peroxodisulfate (KPS), 1-Ethyl-3-(3-(dimethylamino)propyl) carbodiimide hydrochloride (EDC) were purchased from Alfa Aesar (Ward Hill, MA, USA). 1-Hydroxybenzotriazole hydrate (HOBt) was obtained from Fluka (Charlotte, NC, USA). Hydrochloric acid 0.1 M and 1 M (HCl) and sodium hydroxide (NaOH) 0.1 M and 1 M were purchased from Panreac (Chicago, IL, USA). Mesoporous silica particles with a diameter of 500 nm, the dye Rhodamine B (RB), potassium dihydrogen phosphate (KH_2_PO_4_), disodium phosphate (Na_2_HPO_4_) and the solvents dimethylformamide (DMF) and deuterium oxide (D_2_O) were obtained from Sigma-Aldrich (St. Louis, MO, USA). Ultrapure water was provided by means of an ELGA Medica-R7/15 (ELGA Labwater, Woodridge, IL, USA).

### 2.2. Hydrogel Preparation and Rheology Study

Aqueous NaALG-g solutions with various concentrations (5, 7.5 or 10 wt%) were prepared. The polymer solutions were left shaking on a lab shaker (ThermoFisher Scientific, Waltham, MA, USA) at 200 rpm and at 20 °C until homogeneous solutions were obtained. Finally, the pH of the NaALG-g solutions was adjusted at pH = 7.4 using NaOH 1M. A stress-controlled rheometer (AR-2000ex, TA Instruments, New Castle, DE, USA), equipped with a cone and plate geometry (diameter 20 mm, angle 3°, truncation 111 µm), a Peltier plate (TA Instruments, New Castle, DE, USA) that controls precisely the temperature (±0.1 °C) and a solvent trap that prevents concentrations changes due to water evaporation were used to study the rheological profile of the NaALG-g copolymer. The linear viscoelastic regime (LVR) was established in each case by oscillatory strain sweeps. The NaALG-g samples were loaded on the Peltier plate at 40 °C, and the experiments were performed in the LVR.

### 2.3. Preparation of LBL SiO_2_ Particles

**Scheme 1:**
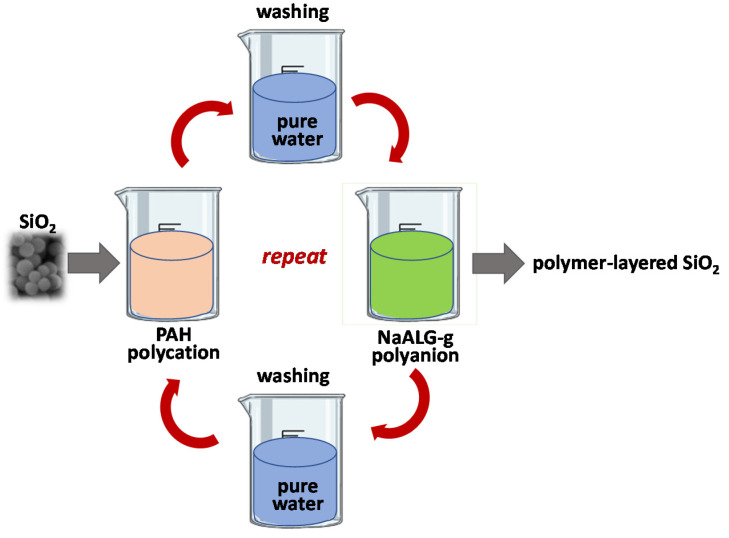
Schematic representation of the LbL method for the fabrication of SiO_2_@polymer hybrid nanoparticles.

Initially, 20 mg of PAH were dissolved in 5 mL of ultrapure water (pH = 6.5). Afterwards, 10 mg of bare powder SiO_2_ particles were added in the PAH solution and left to stir overnight. Next day, the excess of PAH that did not cover the SiO_2_ surface was removed by three centrifugation (3500× *g* rpm, 3 min, Sigma 2K15 Refrigerated Centrifuge, Sigma-zentrifugen, Osterode, Germany)-wash (with 5 mL water, vortex for 5 min, Vortex Shaker, IKA, Wilmington, NC, USA) cycles. The final polymer-coated SiO_2_@PAH particles were centrifuged, separated as a precipitate and dried in the oven (Heraeus vacuum oven, ThermoFisher Scientific, Waltham, MA, USA) at 50 °C. For the coating with the second polymer layer, the SiO_2_@PAH particles were added in an NaALG-g aqueous solution (2 mg NaALG-g dissolved in 4 mL water, pH = 6.5), followed by vortex stirring for 15 min. Again, the unwanted NaALG-g quantity that did not cover the particles surface was removed by three centrifugation-wash cycles, as described above, and the SiO_2_@PAH/NaALG-g particles were obtained by centrifugation and dried in oven at 50 °C. In the same way, two more subsequent PAH (2 mg in 4 mL water, pH = 6.5) and NaALG-g (2 mg in 4 mL water, pH = 6.5) polymer layers covered the particles surface, to prepare the SiO_2_@PAH/NaALG-g/PAH (abbreviated as SiO_2_-single bilayer or hNP) and SiO_2_@PAH/NaALG-g/PAH/NaALG-g (SiO_2_-double bilayer) particles (Scheme 1).

### 2.4. Loading and Release of RB from the LBL SiO_2_ Particles

For the drug release experiments, Rhodamine B dye (RB) was used as a model drug. Initially, an aqueous solution of RB at a concentration of 1.4 mg/mL was prepared and kept in dark. Secondly, 80 mg SiO_2_ were added in 10 mL of the RB solution (mass ratio RB/SiO_2_ = 17.5%). The dispersion was set under stirring for 24 h in dark, and after that period, excess of RB was removed by three centrifugation (3500× *g* rpm, 3 min)-wash (with water, vortex for 5 min) cycles. The RB loaded particles, SiO_2_@RB, were dried in oven at 50 °C. For the preparation of the RB encapsulating LBL silica particles, SiO_2_/RB@/PAH/NaALG-g (SiO_2_-single bilayer) and SiO_2_/RB@PAH/NaALG-g/PAH/NaALG-g (SiO_2_-double bilayer), the same procedure as described in Section 2.3. In this case, the SiO_2_@RB particles were added in the polymer solution instead of bare silica powder. The amount of encapsulated RB in the SiO_2_/RB, SiO_2_/RB@/PAH/NaALG-g and SiO_2_/RB@PAH/NaALG-g/PAH/NaALG-g particles was calculated using a calibration curve of aqueous RB solutions at various concentrations that was constructed with the aim of UV–VIS spectroscopy. Measuring the absorbance of RB at 553 nm of the supernatant solutions removed from the centrifugation/wash cycles and knowing the theoretical added mass of RB, the calculation of encapsulated RB was achieved. Equations (1)–(3) give the theoretical loading (mg/g), the loading amount of RB (mg/g) and the loading efficiency of RB, respectively:

(1) $$\mathrm{Theoretical}\;\mathrm{Loading}\;\left(\frac{\mathrm{mg}}{g}\right)=\frac{\mathrm{initial}\;\mathrm{mass}\;\mathrm{of}\;\mathrm{RB}\;\mathrm{added}\;\mathrm{into}\;\mathrm{particles}\;\left(\mathrm{mg}\right)}{\mathrm{mass}\;\mathrm{of}\;\mathrm{particles}\;\left(g\right)}\;$$

(2) $$\mathrm{Loading}\;\mathrm{Amount}\;\left(\mathrm{mg}/g\right)=\frac{\mathrm{mass}\;\mathrm{of}\;\mathrm{loaded}\;\mathrm{RB}\;\mathrm{into}\;\mathrm{particles}\;\left(\mathrm{mg}\right)}{\mathrm{mass}\;\mathrm{of}\;\mathrm{particles}\;\left(g\right)}\;$$

(3) $$\mathrm{Loading}\;\mathrm{Efficiency}\;\left(\%\right)=\frac{\mathrm{mass}\;\mathrm{of}\;\mathrm{loaded}\;\mathrm{RB}\;\mathrm{into}\;\mathrm{particles}\;\left(\mathrm{mg}\right)}{\mathrm{mass}\;\mathrm{of}\;\mathrm{RB}\;\mathrm{loaded}\;\mathrm{particles}\;\left(\mathrm{mg}\right)}\times 100\%$$

The release of RB from the SiO_2_/RB@/PAH/NaALG-g and SiO_2_/RB@PAH/NaALG-g/PAH/NaALG-g particles was studied with UV–VIS spectroscopy (U-2001 UV–VIS spectrophotometer, Hitachi, Schaumburg, IL, USA). Firstly, a phosphate buffer 10 mM was prepared adding 0.24 g KH_2_PO_4_ and 1.44 g Na_2_HPO_4_ in 1 L ultrapure water. The pH value of the buffer was set at pH = 5.0 or pH = 7.4 using NaOH or HCl 1N. At a next step, 3 mg of SiO_2_/RB@/PAH/NaALG-g or SiO_2_/RB@PAH/NaALG-g/PAH/NaALG-g particles were dispersed in 2 mL of water and transferred in a dialysis membrane (MWCO 12000–14000 Da, (Thermo Fisher, Hampton, NH, USA). In the following, the dialysis membrane was submerged in 10 mL of phosphate buffer 10 mM of pH = 5.0 or 7.4 at 25 °C or 37 °C and shaken at 150 rpm in the dark. At specific timed intervals, the 10 mL of buffer was removed and renewed with 10 mL of fresh buffer. The cumulative release rate (%) of RB was calculated by the absorbance at 533 nm of the buffer samples according to the following Equation (4) and with the aid of calibration curves of RB in phosphate buffer 10 mM at pH = 5.0 and 7.4. All in vitro drug release experiments were carried out in triplicate.

(4) $$\mathrm{RBA}\;\mathrm{cumulative}\;\mathrm{release}\;\mathrm{rate}\left(\%\right)=\frac{\mathrm{Mass}\;\mathrm{of}\;\mathrm{RB}\;\mathrm{released}\;\mathrm{from}\;\mathrm{particles}\;\left(\mathrm{mg}\right)}{\mathrm{Mass}\;\mathrm{of}\;\mathrm{RB}\;\mathrm{loaded}\;\mathrm{into}\;\mathrm{particles}\;\left(\mathrm{mg}\right)}\;\times \;100\%$$

The release data were fitted using the Korsmeyer–Peppas equation (Equation (5)),

(5) $$\frac{M_{t}}{M_{\infty }}={\mathrm{kt}}^{n}$$

where M_t_/M_∞_ is the cumulative release fraction, k is a kinetic constant, t is time in hours, and n is a diffusion or release exponent that indicates the transport mechanism [38,39,40].

### 2.5. Particle/Hydrogel Composite System

A 5 wt% NaALG-g aqueous solution with pH adjusted to 7.4 was prepared, as described in Section 2.2. For the rheology experiments, 3 mg of SiO_2_@/PAH/NaALG-g hybrid nanoparticles (hNP) were encapsulated within 2 mL of the 5 wt% NaALG-g hydrogel, fabricating a nanoparticle/hydrogel composite (hNP@NaALG-g). For the in vitro drug release experiments, 3 mg of RB loaded SiO_2_/RB@/PAH/NaALG-g hybrid nanoparticles (RBhNP) were encapsulated within 2 mL of the 5 wt% NaALG-g solution at 15 °C. The fabricated RB loaded nanoparticle/NaALG-g composite, denoted as RBhNP@NaALG-g, was transferred in a dialysis membrane (MWCO 12000–14000 Da) which in turns was immediately submerged in 10 mL of phosphate buffer 10 mM of pH = 5.0 or 7.4 at 25 °C or 37 °C and shaken at 150 rpm in the dark. At 25 °C, the composite looks like a free-flowing solution while at 37 °C, it seems as an immobile gel. At specific timed intervals, the 10 mL of buffer was removed and renewed with 10 mL of fresh buffer. The cumulative release rate (%) of RB was calculated as described in Section 2.4. All in vitro drug release experiments were carried out in triplicate.

### 2.6. Techniques

A Bruker Avance Iii Hd Prodigy Ascend Tm 600 MHz spectrometer (Billerica, MA, USA) was used to obtain proton nuclear magnetic resonance (^1^H NMR) spectra in D_2_O at 20 °C. Thermogravimetric analysis (TGA) measurements were performed using a Discovery TGA apparatus by TA Instruments (New Castle, DE, USA), under nitrogen atmosphere at a heating rate of 10 °C/min. Zeta potential was carried out on a Malvern Nano Zetasizer analyzer (Malvern, UK) equipped with an He–Ne laser at 633 nm. A Hitachi U-2001 UV–VIS spectrophotometer (Schaumburg, IL, USA) was used to record the UV–VIS spectra. The morphology of the bare and the LbL-coated SiO_2_ particles was observed by transmission electron microscopy (TEM) using a JEM-2100 microscope (JEOL, Tokyo, Japan) operating at 200 kV and by scanning electron microscopy (SEM) using a LEO SUPRA 35VP electron microscope at 15 kV (Cambridge, UK). For TEM measurements, the dried powder samples were dispersed in ultrapure water (0.1 mg/mL), and then, 4 microliters of each dispersion were deposited on carbon grids. The grids were left at room temperature until full evaporation of water. For the SEM measurements, all specimens were sputtered with gold before imaging. A stress-controlled rheometer (AR-2000ex, TA Instruments, New Castle, DE, USA) was used for the rheology experiments. The specific surface area, pore volume and pore size of the bare SiO_2_ particles were calculated applying the Brunauer–Emmett–Teller (BET) and Barrett–Joyner–Halenda (BJH) methods using a Micromeritics Gemini II 2375 Analyzer (Norcross, GA, USA).

## 3. Results

In this work, an alginate-based graft copolymer (denoted as NaALG-g, Scheme S1) constituted of an anionic NaALG backbone, grafted with P(NIPAM_90_-*co*-NtBAM_10_) side chains, was synthesized with the aid of carbodiimide chemistry (detailed synthesis in Supporting Information) [35,41]. The amino functionalized P(NIPAM_90_-*co*-NtBAM_10_) side chains with a 90/10 molar composition of NIPAM/NtBAM monomers and a number average molecular weight Mn = 12,700 g/mol were previously synthesized [35] through free radical polymerization (FRP) (details in Supporting Information) and characterized by acid-base titration, ^1^H NMR and turbidimetry (Table S1). From ^1^H NMR spectroscopy (Figure S1), it was found that the NaALG-g graft copolymer has a grafting density equal to 86, which means that every alginate backbone bears 86 P(NIPAM_90_-*co*-NtBAM_10_) grafting chains (Table S1).

Mesoporous silica particles with a diameter of 500 nm were characterized by BET and BJH methods as well as SEM and TEM microscopy. The characterization results are presented in Table S2. From SEM and TEM microscopy, the silica particles are spherical with a quite smooth surface (Figure S2). The surface of the mesoporous SiO_2_ particles was covered through the LBL method by consecutive oppositely charged polyelectrolyte layers, i.e., the cationic polymer PAH (bearing amino groups) and the anionic polymer NaALG-g (bearing carboxyl groups), in order to prepare nanostructured drug carriers with dual responsiveness (pH- and thermo-). The LbL process used to produce the SiO_2_@polymer hybrids is shown in Scheme 1.

Moreover, a nanoparticle/hydrogel composite has been designed and developed herein, utilizing as gelator the same graft copolymer (NaALG-g) that is used as the anionic polyelectrolyte coat during the preparation of the SiO_2_@polymer hybrid nanoparticles through LBL. This nanoparticle/hydrogel composite is studied by rheology, and its capability as dual responsive drug delivery system is evaluated by in vitro drug release study.

### 3.1. Nanoparticle Fabrication by LbL and Characterization 

**Figure 1:**
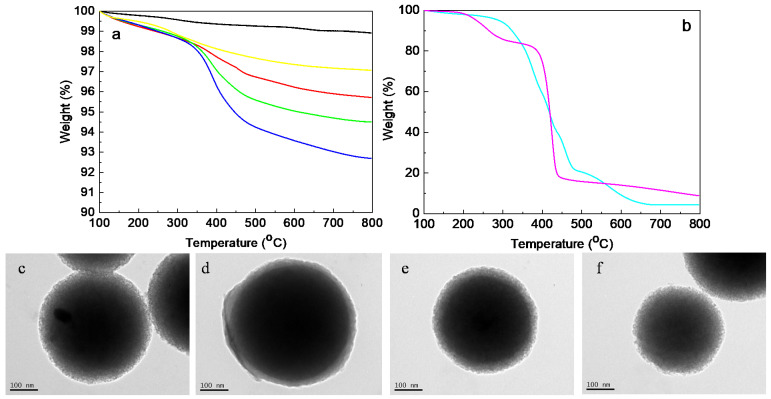
(**a**) Thermogravimetric analysis (TGA) analysis of bare SiO_2_ (black line), SiO_2_@poly(allylamine hydrochloride) (PAH) (yellow line), SiO_2_@/PAH/organic natural polymer sodium alginate (NaALG)-g (red line), SiO_2_@PAH/NaALG-g/PAH (green line) and SiO_2_@PAH/NaALG-g/PAH/NaALG-g (blue line). (**b**) TGA plots of the PAH (cyan line) and NaALG-g (magenta line) polyelectrolytes. TEM images of SiO_2_@PAH (**c**), SiO_2_@PAH/NaALG-g (**d**), SiO_2_@PAH/NaALG-g/PAH (**e**) and SiO_2_@PAH/NaALG-g/PAH/NaALG-g (**f**) particles.

TGA curves of the bare SiO_2_ particles and the as-prepared SiO_2_ particles with successive layers of the polyelectrolytes PAH and NaALG-g are shown in Figure 1a. The bare silica particles exhibit a practically horizontal weight loss curve, with a mass loss of only 1% at 800 °C, indicating that these particles are free of organic remains. For reasons of comparison, the TGA of the polyelectrolytes PAH and NaALG-g are also shown in Figure 1b. The PAH sample displayed a very high weight loss, almost 96% in the range of 400 to 800 °C. A similar behavior was observed for the anionic copolymer NaALG-g which presented a mass loss of 91% for T > 400 °C (Figure 1b). The weight loss behavior of these two polymers is characteristic of the behavior of most polymers and other organic materials. In comparison to the bare SiO_2_ particles and the pure PAH and NaALG-g polymers, the polymer layered SiO_2_ samples exhibited an intermediate mass loss behavior (Figure 1a). For the particles with one polymer layer, SiO_2_@PAH, a weight loss of ~3% is observed in the range of 300 to 800 °C, due to the thermal decomposition of the organic PAH layer that has been added on the particles’ surface. Interestingly, as the number of polymer layers increases, an increase in weight loss of the samples is also seen for temperatures above 300 to 800 °C. Indeed, for the SiO_2_@PAH/NaALG-g particles (two polymer layers), a weight loss of ~4.3% can be seen, while for the SiO_2_@PAH/NaALG-g/PAH (three polymer layers) and SiO_2_@PAH/NaALG-g/PAH/NaALG-g (four polymer layers) particles, a weight loss of 5.5% and 7.3% is observed, respectively. These results undeniably confirm that the consecutive PAH and NaALG-g polyelectrolyte layers have effectively covered the surface of the SiO_2_ nanoparticles. 

Figure 1c–f show typical TEM images of the as-prepared SiO_2_@polymer nanoparticles. Compared to bare SiO_2_ particles which were spherical particles with a quite smooth edge, as seen in the inset of Figure S2, the morphology of the LBL polymer coated SiO_2_ particles is different. The surface of all these SiO_2_@polymer particles displays an increased roughness, proving the successful deposition of successive PAH and NaALG-g polymer coatings onto the SiO_2_ surface.

**Figure 2:**
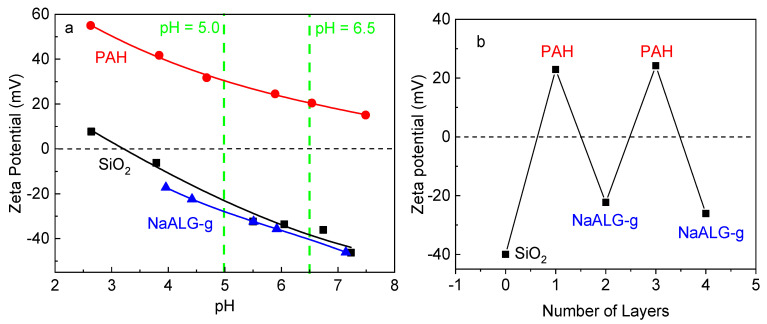
(**a**) pH-dependent zeta potential of bare SiO_2_ (black squares), PAH (red circles) and NaALG-g (blue triangles). All sample solutions have a concentration of 3 mg/mL, and the pH was adjusted using HCl 0.1 M or NaOH 0.1 M. (**b**) Evolution of zeta potential during the sequential deposition of the different layers on the surface of silica particles in aqueous solution (3 mg/mL, pH = 6.5).

As already mentioned in the experimental part (Section 2.3), the addition of the sequential polymer coatings on the silica particles was performed using aqueous PAH and NaALG-g polymer solutions with a value of pH = 6.5. This condition was chosen to achieve as high as possible interaction between the silica surface and the PAH and NaALG-g polymers due to ionic bonds, which will in turn lead to high surface covering efficiency. In Figure 2a, the pH dependence of the zeta potential values of bare SiO_2_ aqueous dispersions and the aqueous PAH and NaALG-g polymer solutions are presented. The SiO_2_ particles have a slight positive zeta potential at pH < 3.0, which becomes negative as pH increases. In fact, above pH = 5.0, the zeta potential of SiO_2_ particles takes values of around −30 mV, and it reaches the value of −46 mV at around pH = 7.5, forming quite stable aqueous dispersions. For the PAH solutions, the zeta potential obtains positive values throughout all the pH range studied, as expected due to the cationic nature of this polyelectrolyte. The zeta potential has a value of 55 mV at acidic environment, while it decreases with pH increase to reach the value of 15 mV at pH ~ 7.5. On the other hand, the NaALG-g graft copolymer, an anionic polyelectrolyte that bears carboxylic groups on its NaALG backbone, exhibits negative zeta potential values in the range of pH studied (pH = 4.0 to pH = 7.5). The zeta potential of NaALG-g becomes more negative with increasing pH due to the ionization of the carboxyl groups (–COO^−^). Electrophoresis was also used to monitor the consecutive coating of the SiO_2_ particles surface with polymer layers. In Figure 2b, the evolution of zeta potential of the SiO_2_ particles surface after each polymer layer is presented. At first, the bare silica particles display a negative value of −40 mV at pH = 6.5, in accordance with the findings in Figure 2a, discussed previously. After the addition of the PAH layer, the zeta potential of the particles (SiO_2_@PAH) takes a value of +23 mV, owing to the positive charge of the amine groups of PAH that dominate on the negative charge of the bare silica surface, indicating the successful deposition of a PAH layer on the SiO_2_ surface. In the following, when the next NaALG-g polymer is added, the zeta potential of the particles (SiO_2_@PAH/NaALG-g) alters and now obtains the negative value −22 mV, due to the ionized –COO^−^ groups of NaALG-g, verifying again the effective deposition of a second polymer layer on the silica surface. At a next step, when an extra PAH layer is added, the zeta potential of the particles (SiO_2_@PAH/NaALG-g/PAH) obtains a positive value +24 mV, again signifying the successful deposition of another PAH layer on the particle. Finally, after adding another layer of NaALG-g, the particles (SiO_2_@PAH/NaALG-g/PAH/NaALG-g) exhibit a zeta potential *−*26 mV, confirming the effective covering of the particles surface with the anionic NaALG-g polymer. Thus, through electrophoresis, it can be concluded that the SiO_2_ nanoparticles are efficiently coated by alternating PAH and NaALG-g layers through the LBL technique, due to ionic interactions of the oppositely charged successive layers.

### 3.2. NaALG-g Rheological Properties: Thermo-Induced Gelation, Injectability

Nanocarriers loaded with payloads encapsulated within an injectable hydrogel constitute another strategy to design controlled sustainable DDS [42,43,44,45,46,47,48,49,50]. One of the benefits of this strategy is that the nanocarriers are retained in the targeting point and release the therapeutics sustainably, due to the very high viscosity exhibited within the hydrogel environment.

**Figure 3:**
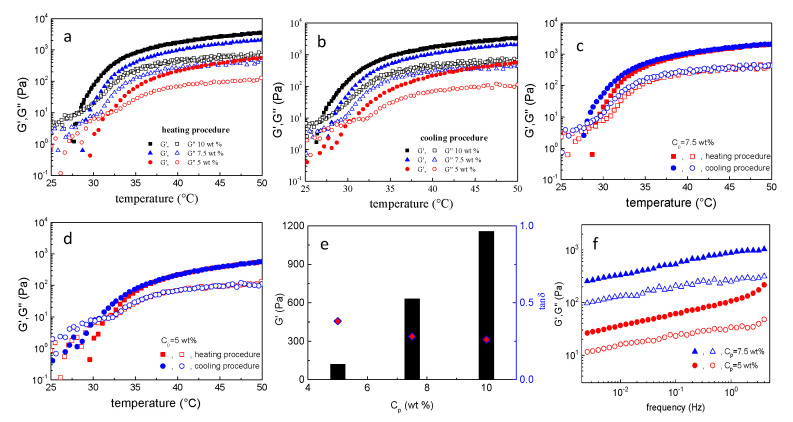
(**a**) Storage modulus G’ (solid symbols) and loss modulus G’’ (open symbols) at f = 1 Hz, γ = 0.1% and rate of 1 °C/min at different C_p_ as indicated: (a) heating procedure (**b**) cooling procedure, (**c**) cooling/heating cycle for C_p_ = 7.5 wt% and (**d**) for C_p_ = 5 wt%, (**e**) G’ and tanδ versus C_p_ at 37 °C extracted from the data of plot (**a**), (**f**) frequency dependence of G’ (solid symbols) and loss modulus G’’ (open symbols) at 37 °C for C_p_ = 7.5 wt% (triangles) and C_p_ = 5 wt% (circles) for NaALG-g aqueous solutions.

Herein a nanoparticle/hydrogel composite has been also developed utilizing as gelator the same graft copolymer with the one used to prepare the SiO_2_@PAH/NaALG-g hybrid nanoparticles. To evaluate the thermo-induced gelation of the bare NaALG-g gelator, temperature ramp oscillatory experiments were firstly conducted in three aqueous formulations regulated at pH = 7.4 and differing in the polymer concentration (C_p_). In Figure 3, the storage (G’) and the loss (G”) moduli are plotted versus temperature either by heating (Figure 3a) or cooling (Figure 3b) ramp, applying a rate of 1 °C/min in all cases. The data demonstrate a G’/G” crossover at a critical temperature, namely T_gel_, above which a 3D network forms, due to the intermolecular hydrophobic association of the LCST type P(NIPAM_90_-*co*-NtBAM_10_) grafted chains (stickers) of the gelator. We note that T_gel_ depends on the applied frequency, implying that it is an apparent value [51]. As can be observed, the T_gel_ is slightly sensitive to C_p_. Particularly it decreases upon increasing C_p_, i.e., from 31.7 °C at C_p_ = 5 wt% to 28.5 °C at C_p_ = 10 wt%, in agreement with analogous thermoresponsive physical networks [52]. This effect is probably due to the cloud point suppression (controlling the solubility and in turn the association capability of the grafting chains), that is influenced by C_p_, resulting to hydrophobic crosslinking at lower temperature. Alternatively, the increase of G’ (again C_p_-depedent, see below) likely crosses G’’ at slightly lower T. Similar trends were observed for the heating and cooling procedures (Table S3). However, there is a slight hysteresis between them.

In Figure 3c,d, the cooling/heating cycles for two concentrations are demonstrated, showing some hysteresis which is more pronounced in the vicinity of the G’/G” crossover, affecting slightly the T_gel_. At high temperatures, the moduli seem to coincide. The hysteresis (ΔT_gel_) is weakening upon decreasing concentration and from 1.5 °C at C_p_ = 10 wt% becomes 0.6 °C for C_p_ = 5 wt% (Table S3). More importantly, C_p_ affects the magnitude of the moduli as expected. As it is well known, the storage modulus G’ is controlled by the number density (*n*) of the elastically active chains (segments between crosslinks), according to the rubber elasticity theory through the equation *G = nkT* (where *G* is the elastic modulus, *k* is the Boltzmann’s constant, and *T* the absolute temperature in Kelvin) [51,52,53]. Obviously the higher the C_p_, the higher the moduli (Figure 3e), which allows tuning of the magnitude of the elastic modulus and viscosity of the hydrogels through the gelator concentration, which is important for targeting applications.

Finally, oscillatory frequency sweeps were performed in the linear viscoelastic regime at 37 °C for the samples with C_p_ of 7.5 and 5 wt%. As seen in Figure 3f, G’ is higher than G” in the entire frequency range investigated for both gelator concentrations. The terminal relaxation zones (G’/G” crossover) are not visible even at low frequency of 2 × 10^−3^ Hz, implying long terminal relaxation times and in turn slow dynamics of the network formed at 37 °C through the thermo-induced association of the pendant sticky P(NIPAM_90_-*co*-NtBAM_10_) chains.

**Figure 4:**
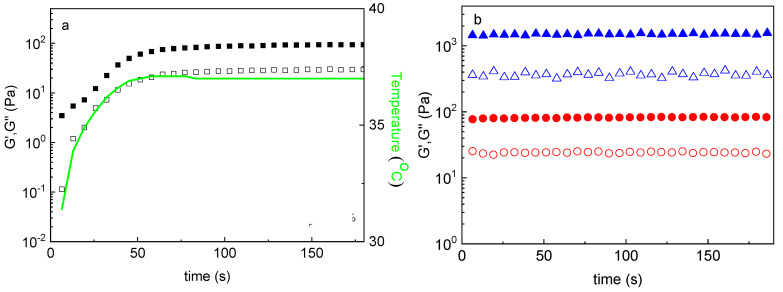
Storage G′ (closed symbols) and loss G″ (open symbols) moduli as a function of time (**a**) of the 5 wt% NaALG-g polymer aqueous solution without nanoparticles (the green line implies the progression of temperature from 25 °C to 37 °C) and (**b**) of the 7.5 wt% (triangles, blue) and of the 5 wt% (circles, red) graft copolymer solutions equilibrated at 37 °C.

The injectability of the system was evaluated designing the following experiment. The formulation was equilibrated at 25 °C, where it behaves as sol (G’ < G”, see Figure 3) and subsequently was subjected to a temperature jump at 37 °C, applying time sweep under 0.1% strain amplitude (linear viscoelastic regime) without ticking the equilibration box in the instrument. As observed in Figure 4a, this procedure allows to capture the evolution of the moduli with temperature up to equilibration at 37 °C, which was established after 75 s. The data indicate an instantaneous temperature response of the material, as manifested by the formation of the network (G’ > G’’) immediately above T_gel_. Moreover, the evolution of moduli follows the evolution of temperature, showing perfect response. These results suggest that the hydrogel can be formed in situ after injection, fulfilling the requirements of the minimally invasive operation. The same hydrogel behavior was also observed for the higher C_p_ of 7.5 wt% (Figure 4b). Note the data in this figure were obtained after temperature equilibration at 37 °C, by ticking the equilibration box. In this case the acquisition of the data starts after temperature equilibration.

**Figure 5:**
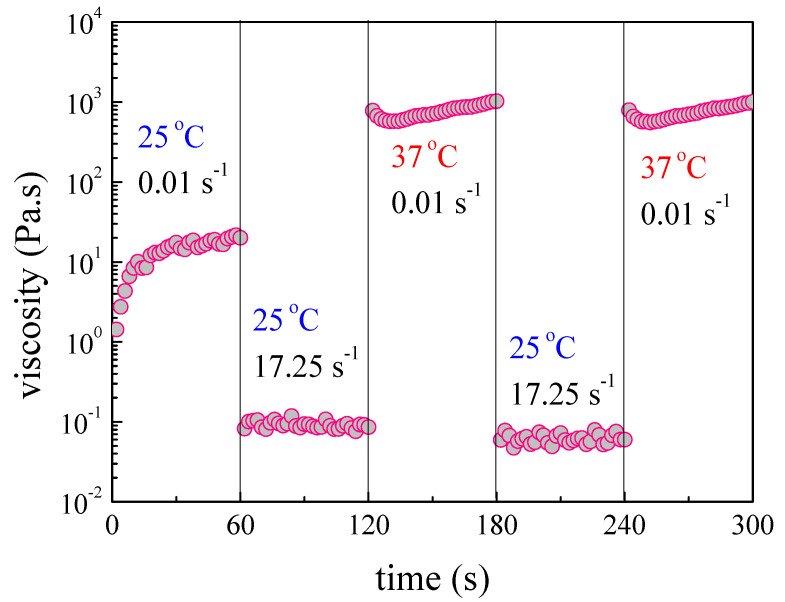
Shear viscosity versus time of a 5 wt% NaALG-g aqueous solution without the presence of nanoparticles at different shear rates 0.01 s^−1^ (at 25 °C), 17.25 s^−1^ (at 25 °C) and 0.01 s^−1^ (at 37 °C) and conversely.

Finally, steady state shear viscosity experiments were conducted simulating conditions of injection through a 28-gauge needle syringe. Particularly, time dependent shear viscosity was measured in consecutive steps of 60 sec duration and under different conditions, namely, 25 °C/γ = 0.01 s^−1^, 25 °C/17.25 s^−1^ and 37 °C/γ = 0.01 s^−1^ [35]. Figure 5 demonstrates the results, indicating several effects: shear thinning responsiveness as the viscosity drops instantly, more than two orders of magnitude upon applying a high shear rate of 17.25 s^−1^ and simultaneously shear/thermo thickening effect by rising the temperature at 37 °C and decreasing the shear rate again to 0.01 s^−1^ (approach of zero-shear conditions) simulating the immobilization of the hydrogel at physiological temperature. Importantly, the viscosity under high shear (injection conditions) is of the order of 0.1 Pa·s which suggests very good injectability.

### 3.3. Nanoparticle/Hydrogel Composite

**Figure 6:**
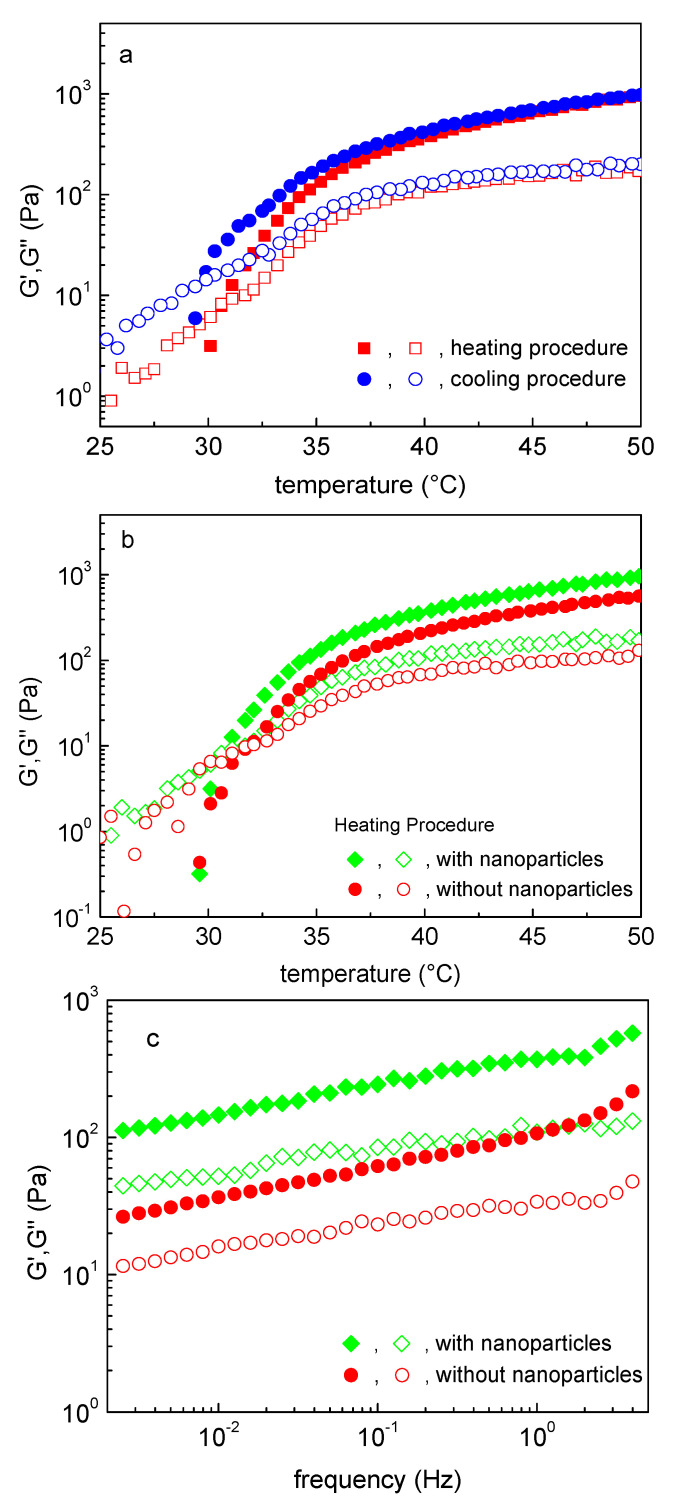
Temperature dependence of storage modulus G’ (solid symbols) and loss modulus G’’ (open symbols) (**a**) heating/cooling cycle of 5 wt% NaALG-g graft copolymer aqueous solution with hNP, (**b**) at heating procedure at of 5 wt% polymer aqueous solutions with hNP (rhombus, green) and without (circle, red). The heating/cooling procedures were performed at 1 Hz and strain amplitude of 0.1% at a rate of 1 °C/min. (**c**) Frequency sweep at 37 °C of storage G′ (closed symbols) and loss G″ (open symbols) moduli of 5 wt% NaALG-g aqueous solutions with hNP (rhombus, green) and without hNP (circle, red).

SiO_2_@PAH@NaALG-g hybrid nanoparticles (hNP) were encapsulated within the 5 wt% NaALG-g hydrogel, fabricating a nanoparticle/hydrogel composite (hNP@NaALG-g). Rheological investigation of the composite was performed to evaluate the influence of the presence of the hNP to the hydrogel properties of the composite. In Figure 6a, the oscillatory shear temperature ramp is depicted for a cooling/heating cycle showing similar behavior with the bare hydrogel, that is, a sol–gel transition upon heating, with a T_gel_ at 30.7 °C, just one degree lower than that of the hydrogel without hNP (see Table S3). More importantly, the moduli are higher for the composite hydrogel in the entire temperature range above T_gel_, as can be observed clearly in Figure 6b, implying that the hNP contribute to the formed polymeric network as its elasticity was reinforced. This result is confirmed by oscillatory frequency sweep at 37 °C as demonstrated in Figure 6c. Again, a gel like behavior (G’ > G”) can be seen in the entire frequency range measured without any crossing at low frequencies for the hNP@NaALG-g composite system. However, the elastic modulus of the composite is about three times higher than that of the bare hydrogel. Considering the fact that the outer layer of the SiO_2_@PAH/NaALG-g NPs is constituted of the same thermo-responsive NaALG-g, it is reasonable to contribute to the physical crosslinking of the gelator through the common P(NIPAM_90_*-co-*NtBAM_10_) grafted chains thermo-induced association, increasing therefore the number density of crosslinks, which is reflected to the G’ augmentation.

**Figure 7:**
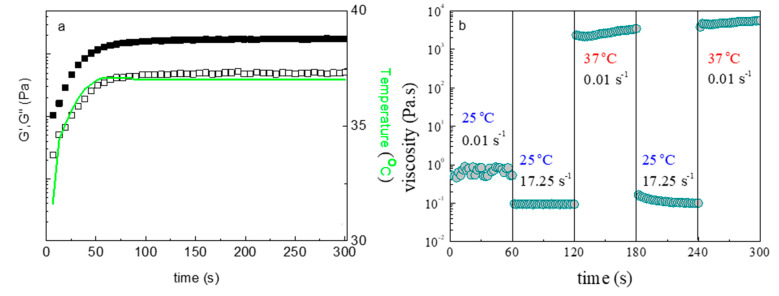
(**a**) Storage G′ (closed symbols) and loss G″ (open symbols) moduli as a function of time after temperature jump from 25 to 37 °C (the green line indicates the change of temperature towards equilibration at 37 °C) and (**b**) shear viscosity versus time of at different shear rates 0.01 s^−1^ (at 25 °C), 17.25 s^−1^ (at 25 °C) and 0.01 s^−1^ (at 37 °C) and conversely for the hNP@NaALG-g composite system (C_p_ = 5 wt%).

The injectability of the system was evaluated by similar experiments as described previously for the bare hydrogel (Figure 4 and Figure 5). The hNP@NaALG-g responds instantly to stepwise increase of temperature from 25 °C to 37 °C by fast gel formation at physiological temperature (Figure 7a). Furthermore, shear thinning response at room temperature shows instantaneous drop of the apparent viscosity at 0.1 Pa.s and shear/thermo response upon concurrent decreasing shear rate and increasing temperature at 37 °C. Interestingly, the viscosity at room temperature of the composite is lower about one order of magnitude from the one of the bare hydrogel, implying that in the sol state (non-associated NaALG-g), the presence of the negatively charged nanoparticles, mixed with the negatively charged polymer, facilitates better flow of the formulation.

In conclusion, the hNP@NaALG-g composite system exhibits a sol–gel transition at a temperature between room and body temperature with very good injectability. Moreover, the composite hydrogel exhibits better flow at room temperature and notable mechanical reinforcement at body temperature, favoring better immobilization of the hNP in the position of injection.

### 3.4. Controlled RB Release In Vitro 

A hydrophilic model drug, RB, was chosen to explore drug release from the hybrid polymer formulations designed in this article. According to the experimental procedure and the Equations (1)–(3) described in Section 2.4, the theoretical RB loading (mg/g), the loading amount of RB (mg/g) and the loading efficiency of RB were calculated to be 175.0 mg/g, 51.25 mg/g and 4.85%, respectively, for the RB loaded particles, SiO_2_/RB. The RB loaded silica particles were further coated by PAH and NaALG-g polymer coats by the LBL method. The final SiO_2_/RB@PAH/NaALG-g and SiO_2_/RB@PAH/NaALG-g/PAH/NaALG-g particles had an RB Loading Efficiency of 1.77% and 1.13%, respectively. The decrease of the RB Loading Efficiency in the hybrid SiO_2_/RB@PAH/NaALG-g and SiO_2_/RB@PAH/NaALG-g/PAH/NaALG-g particles, compared to the SiO_2_/RB particles, is attributed to the subsequent wash/centrifugation cycles after each polymer coat deposition on the prefabricated SiO_2_/RB particles during the LBL process, which lead to inevitable RB loss from the coated silica nanoparticles.

**Figure 8:**
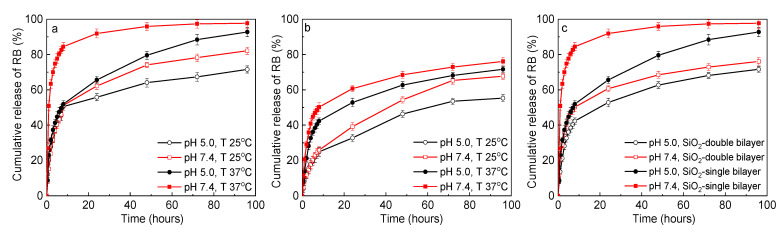
Release of RB at different pH and temperatures from the (**a**) SiO_2_-single bilayer (SiO_2_@PAH/NaALG-g) and (**b**) SiO_2_-double bilayer particles (SiO_2_@PAH/NaALG-g/PAH/NaALG-g). (**c**) For the sake of comparison, the data of the RB release from the SiO_2_-single bilayer (Figure 8a) and the SiO_2_-double bilayer (Figure 8b) particles at pH = 5.0, pH = 7.4 and at 37 °C are also presented. Each point is the mean of three independent measurements, and bars represent standard deviation of means.

The pH- and thermo- responsive in vitro drug release of RB from the SiO_2_@polymer hybrid nanoparticles as well the hNP@NaALG-g composite system was investigated in PB solutions at fixed pH = 5.0 and pH = 7.4 as well as at room temperature (25 °C) and at body temperature (37 °C). In Figure 8a,b, the RB release profiles of the SiO_2_@PAH/NaALG-g (SiO_2_-single bilayer) NPs and the SiO_2_@PAH/NaALG-g/PAH/NaALG-g (SiO_2_-double bilayer) NPs are presented, respectively. A two-step (fast/slow) release kinetics is exhibited in all cases. In the first ten hours of the release process, fast release of RB occurs which might be attributed to the RB molecules trapped within the polymeric layers during the LbL covering procedure. The followed slow step should be due to the release of the RB molecules encapsulated within the mesoporous silica NPs. Obviously, the release kinetics depend on the incubation conditions of pH and temperature with the faster overall release occurring at pH 7.4 and 37 °C (physiological conditions) in both single- and double-bilayer covered NPs. Concerning the SiO_2_-single bilayer NPs, at 37 °C (Figure 8a), it can be seen that the release of RB is higher at the physiological pH (pH 7.4) than the RB release at the slightly acidic environment of pH = 5.0, i.e., 92% versus 66% after 24 h. The temperature also seems to control the release kinetics. At pH = 7.4 the RB release is faster at 37 °C with respect to room temperature (25 °C), i.e., 92% versus 62% after 24 h. However, the temperature effect seems week at pH = 5.0 (66% versus 56% 24 h) and even weaker the first ten hours (fast step), i.e., 51.9% versus 50.5%. Nevertheless, after 4 days overall drug delivery, faster RB release occurs at higher pH and/or temperature in both systems (either single- or double-bilayer NPs).

**Scheme 2:**
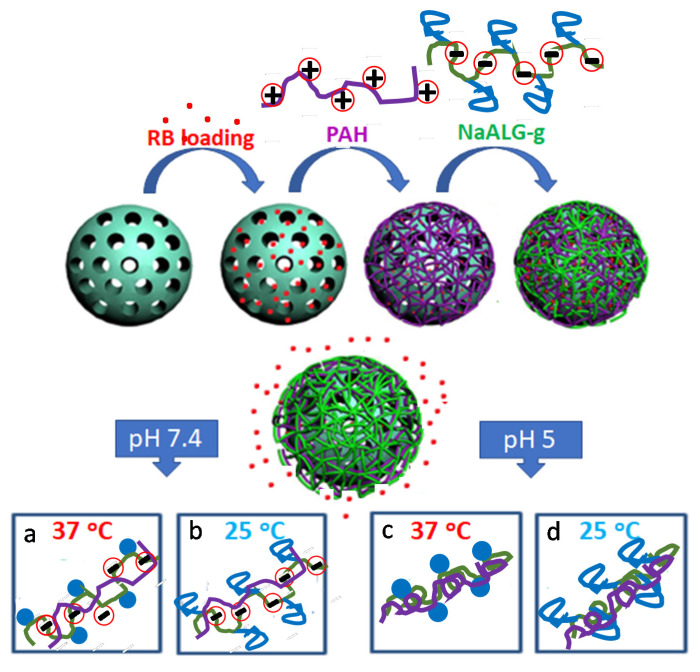
Schematic representation of RB loaded in MSN and LbL (single bilayer) hNP formation along with its membrane structure at various pH/temperature conditions during RB release: open structure due to the excess negative charges of Alginate with side chains adopting either globule conformation (**a**) or coil conformation (**b**); closed structure due to charge compensation, with side chains either globule conformation (**c**) or coil conformation (**d**). (PAH purple, NaALG green, PNIPAM blue). For clarity, charges and grafting chains of Alginate at the surface of MNP have been omitted.

In order to understand the above results, we should look at the molecular level of the polymer chain conformations and the interactions between the different chains covering the MSNs at the various conditions investigated. There are two main effects: the ionic interactions between the oppositely charged macromolecules, governed by pH, and the LCST-type coil-globule transition of the PNIPAM-based grafting chains on the alginate, governed by temperature (Scheme 2). Concerning the effect of pH, it could be understood with the aid of the zeta potential study presented in Figure 2a. As can be observed, at pH = 5.0, both PAH and NaALG-g polymers exhibit about equal positive (+30 mV) and negative (−28 mV) zeta potential, respectively. We assume that the oppositely charged polyelecrtrolytes, adsorbed on the silica NPs, exhibit maximum ionic interactions, converting thus the polymer bilayer coat surface more compact which act as effective pore keeper, delaying thus the release rate of RB. On the contrary, at pH = 7.4, the negative charges of the NaALG-g prevail (−40 mV versus +20 mV), leading to repulsive forces between the polymer chains and thus more extended chain conformations, which swell the bilayer pores, facilitating RB diffusion. 

As far as the temperature effect is concerned, the RB release is faster at 37 °C rather than 25 °C; that should be attributed to the LCST-type conformational transition of the P(NIPAM_90_-*co*-NtBAM_10_) grafted chains of the NaALG-g graft copolymer. Indeed, the grafting chains (85 per alginate) exhibit an LCST of 25 °C (onset of cloud point) [35] above which they lose their high hydrophilicity in the aqueous medium, adopting a compact conformation at 37 °C. Therefore, the shrinkage of the graft copolymer chains that cover the silica particles surface leads to a weakened pore keeper effect (increasing the pore bilayer size), and therefore, RB diffuses out easier from the hNPs at body temperature. The temperature effect is more pronounced at pH = 7.4 due to the repulsive interactions along and among the NaALG-g chains resulting to a more open structure of the bilayer, as discussed above. Overall, the increase of pH from 5 to 7.4 and simultaneously the temperature from 25 °C to 37 °C, both changes contribute to an easier penetration of the bilayers from the payload, facilitating maximum drug release rate.

The same trend in the release profile is observed from the silica nanoparticles covered with four successive layers, SiO_2_@PAH/NaALG-g/PAH/NaALG-g (SiO_2_-double bilayer), as demonstrated in Figure 8b. It is worth mentioning that under all conditions studied (different pH and temperatures), this system exhibited a slower release rate of RB, compared to the SiO_2_-single bilayer system presented in Figure 8a. The influence of the number of bilayers can be better seen in Figure 8c, where the data for both systems at pH = 5.0, pH = 7.4 and at 37 °C are shown. The released amount of RB after 4 days from the SiO_2_-double bilayer NPs is about 77% of that released from the SiO_2_-single bilayer NPs, regardless of pH. This effect is attributed to a thicker polymer coat that acts as a more efficient pore keeper, disabling the fast diffusion and slowing down the release of RB. It can be undoubtedly concluded that the number of the consecutive polymer layers that cover the silica NPs surface through the LBL technique is another important factor controlling the drug release rate.

**Scheme 3:**
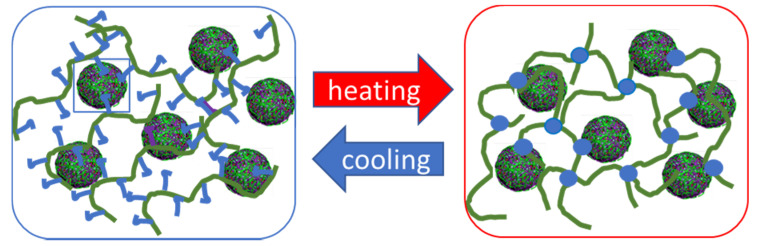
Schematic representation of the thermo-responsive behavior of the hNP/hydrogel composite. Upon heating a network form, constituted of Alginate chains (green lines), crosslinked by hydrophobic association of the side chains (blue spheres), the hNPs contribute to crosslinking. The process is reversible. For clarity, the negative charges of Alginate have been omitted.

**Figure 9:**
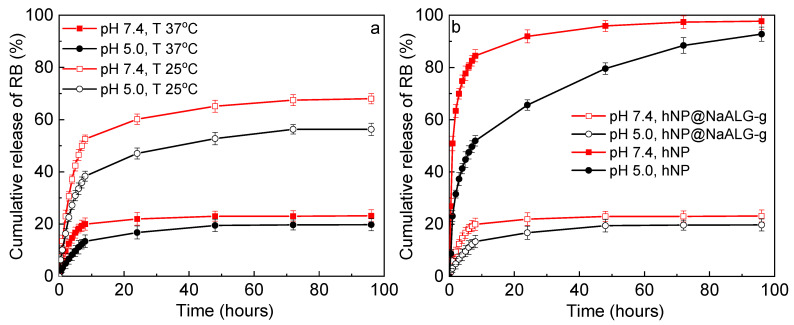
(**a**) Release of RB at different pH and temperatures from the composite hNP@NaALG-g formulation for a 5% wt NaALG-g hydrogel. (**b**) For the sake of comparison, the data of the RB release, at pH 5.0, pH 7.4 and at 37 °C, from the hNP particles (single bilayer: SiO_2_@PAH/NaALG-g, from Figure 8a) and the hNP@NaALG-g composite hydrogel (from Figure 9a) are also presented. Each point is the mean of three independent measurements, and bars represent standard deviation of means.

The release of RB from the hNP/hydrogel composite material (RBhNP@NaALG-g) was further investigated (Figure 9). In this case, the RB loaded hNP nanoparticles (single bilayer) were incorporated in a 5% wt NaALG-g aqueous solution at 15 °C in a dialysis membrane and immediately submerged in 10 mL of phosphate buffer of pH = 5.0 or 7.4 at 25 °C or 37 °C. As seen in Figure S3, where vial inversion test was performed, the copolymer NaALG-g at room temperature (T 25 °C) and at a concentration of 5% wt forms a free-flowing viscous aqueous solution. Upon increasing temperature at T = 37 °C, the solution loses its mobility and becomes a free-standing hydrogel due to hydrophobic association of the P(NIPAM_90_-*co*-NtBAM_10_) grafted chains (Scheme 3 and Figure 6). As seen in Figure 9a, the composite system displays a higher RB diffusion at higher pH but at lower temperature, regardless of pH. The temperature effect is now inverse to that observed for the neat hNPs. At 25 °C, the release of RB from RBhNP@NaALG-g composite at 4 days is 68% and 56% for pH = 7.4 and pH = 5.0, respectively, much lower than the RB release from the neat RBhNPs at the same experiment conditions (i.e., 82% for pH = 7.4 and 72% for pH = 5.0, Figure 8a). Obviously, at room temperature, the non- associated NaALG-g only forms a viscous solution with a shear viscosity slightly lower than 1 Pa (Figure 7b) but considerably higher than that of the water medium (~10^−3^ Pa), which apparently reduces the RB diffusion. At 37 °C, due to the hydrogel formation, as manifested by the dramatic increase of the shear viscosity of the medium (about 2 × 10^3^ Pa, see Figure 7b), even lower RB diffusion from the composite hydrogel resulted. The thermo-induced hydrogel formation is now the determining factor which overcompensates the temperature effect observed for RB release through the neat hNPs.

Evidently, the most profound difference was observed at 37 °C. For the sake of comparison, the results of RB release, from the RBhNP particles (Figure 8a) and the RBhNP@NaALG-g composite (from Figure 9a) at pH = 5.0, pH = 7.4 and at 37 °C are presented. As seen, the hNP@NaALG-g composite has a very low RB release of 23% (at pH = 7.4) and 20% (at pH 5.0) at 96 h, compared to the high release of 98% (at pH = 7.4) and 93% (at pH = 5.0) of RB from the neat hNPs. These findings indicate that the 3D structure of the NaALG-g hydrogel was able to drastic slow down the release of RB [38,54].

In order to investigate the mechanism involved in the drug diffusion process at 37 °C, the early portion of the release data of Figure 8c and Figure 9b was fitted to the Korsmeyer–Peppas (namely also as Ritger–Peppas) equation (Equation (5)). In this equation, *k* is the release constant, which is proportional to the diffusion coefficient *D* (*k*~*D*^1/2^), and *n* is the diffusional exponent showing the mechanism of drug release, i.e., n ≤ 0.5 describing Fickian diffusion-controlled release, 0.5 < n < 1, representing anomalous (non-Fickian) transport of drugs, and n = 1 indicating the case-II release of drugs [55]. The fitting of the RB fraction released per time for the RBhNP particles and the RBhNP@NaALG-g composite hydrogel at pH = 5.0, pH = 7.4 and at T 37 °C are plotted in Figure S4, and the results are summarized in Table 1. The linearity of the data for the time range of 0–8 h is good (R^2^ between 0.999 and 0.981), confirming the validity of the kinetic model (Equation (5)) for the early stage of RB release. Concerning the *k* values, correlated with the release rate of RB, they increase about twofold upon increasing pH from 5.0 to 7.4 in the neat hNPs as well in the hNP/hydrogel composites, demonstrating an increase of the RB release rate with increasing pH. More importantly, at the same pH the *k* values decrease, about one order of magnitude, when RB releases from the hNP in sink conditions than when it releases encapsulated in the hydrogel, suggesting noticeable slowdown of the RB release rate through the hydrogel. This trend is valid also from single to double bilayer covered hNPs (Table 1), i.e., the increase of layers slowdowns the release rate. Furthermore, the change of the values of *n* reveals change of the release mechanism from Fickian diffusion-controlled to anomalous (non-Fickian) transport of drug form neat hNP to hNP/hydrogel composites respectively, irrespective of pH. The same trend was observed when passing from single to double bilayered hNP only at pH = 5.0 (Table 1). Overall, the RB release from the composite hydrogel is consistent with sustained release of payloads, following anomalus non-Fickian kinetics in agreement with analogous behavior reported for hydrogel DDSs [48,56,57].

## 4. Conclusions

In the present paper, we demonstrate the ability of an Alginate, highly grafted by 86 thermoresponsive P(NIPAM_90_-*co*-NtBAM_10_) chains, to be used as a gate keeper of MSNs to prepare hybrid nanoparticles for drug delivery platforms. Moreover, the same alginate graft copolymer was utilized as gelator to prepare a nanoparticle/hydrogel composite system with sustained drug delivery capabilities.

The LbL technique was used to prepare the hNPs by successive deposition of PAH and NaALG-g weak polyelectrolytes which were stabilized by ionic interactions between their oppositely charged moieties. RB was used as the hydrophilic model drug to evaluate the release capability of the system. Three main factors govern the RB release from the hNPs: (1) the number of PAH/NaALG-g bilayers, i.e., the increase of the number of bilayers results to a slowdown of the RB release; (2) pH, affecting the interactions of the oppositely charged polyelectrolyte partners; and (3) temperature, affecting the conformation of the P(NIPAM_90_-*co*-NtBAM_10_) grafting chains. The two latter environmental factors affect the permeability of the layered membrane of the hNPs, i.e., the higher the pH and/or temperature are, the faster the RB release.

By encapsulating the RB loaded hNPs within a 5 wt% NaAlg-g- P(NIPAM_90_-*co*-NtBAM_10_) solution, a thermo-induced injectable composite hydrogel was fabricated, responsive again to pH. In this DDS, temperature is the main determining factor of the RB release. Thanks to the thermo-induced gelation at physiological temperature, the system exhibits dramatic slowdown of the release kinetics transformed hence to a sustainable DDS. The composite hydrogel, with tunable rheological properties, exhibits excellent injectability and seems promising to be evaluated for biomedical applications.

## Figures and Tables

**Table 1:** Fitting parameters obtained by applying the Korsmeyer–Peppas model (Equation (5)) to the RB release data at 37 °C.

Sample	Analysis in the Time Range: 0–8 h		
	k_1_ (hr^-n^)	n	R^2^
pH 5.0, RBhNP (SiO_2_-single bilayer)	0.238	0.386	0.994
pH 7.4, RBhNP (SiO_2_-single bilayer)	0.526	0.239	0.981
pH 5.0, RB/SiO_2_-double bilayer	0.029	0.547	0.983
pH 7.4, RB/SiO_2_-double bilayer	0.029	0.435	0.985
pH 5.0, RBhNP@NaALG-g	0.029	0.738	0.999
pH 7.4, RBhNP@NaALG-g	0.056	0.656	0.984

## Data Availability

The data presented in this study are available on request from the corresponding author.

## Supplementary Materials

The following are available online at https://www.mdpi.com/article/10.3390/polym13081228/s1. Scheme S1: The structure of the NaALG-g-P(NIPAM_90_*-co-*NtBAM_10_) graft copolymer; Figure S1: ^1^H-NMR spectra of NaALG, the side chains P(NIPAM_90_*-co-*NtBAM_10_) and the graft copolymer NaALG-g; Table S1: Molecular characteristics of the polymers; Table S2: Characteristics of the SiO_2_ particles; Figure S2: SEM photo of the bare SiO_2_ particles. Inset: TEM image of the bare SiO_2_ particles; Table S3: T_gel_ at heating or cooling cycles and hysteresis (ΔT_gel_) for the copolymer with or without the SiO_2_@PAH/NaALG-g hybrid nanoparticles (hNP); Figure S3: Photos of the NaALG-g: aqueous solution at 25°C and free-standing hydrogel at 37 °C; Figure S4: Korsmeyer–Peppas fitting (Equation (5)) of RB release data: (a) for the SiO_2_-single bilayer (hNP) and the SiO_2_-double bilayer particles at pH 5.0, pH 7.4 and (b) for the hNP particles and the hNP@NaALG-g composite hydrogel at pH 5.0, pH 7.4, all at T 37 °C. The vertical line denotes 8 h.
