# Supplementary material for: pH/Thermo-Responsive Grafted Alginate-Based SiO2 Hybrid Nanocarrier/Hydrogel Drug Delivery Systems
Source: Polymers (Basel). 2021 Apr 10;13(8):1228. doi: 10.3390/polym13081228 (PMC8069398; doi:10.3390/polym13081228)
Supplement: Supplementary file 1 [file polymers-13-01228-s001.pdf]

pH/Thermo-Responsive Grafted Alginate-based SiO_2 hybrid Nanocarrier/Hydrogel Drug Delivery Systems

Nikolaos Theodorakis, Sofia-Falia Saravanou, Nikoleta-Paraskevi Kouli, Zacharoula Iatridi*, Constantinos Tsitsilianis*

Department of Chemical Engineering, University of Patras, 26500 Patras, Greece

Email addresses

nikolastheo96@hotmail.com

faliasaravanou@hotmail.com

nikoleta.li.paraleta@gmail.com

iatridi@upatras.gr

ct@chemeng.upatras.gr

Synthesis of the amino functionalized P(NIPAM$_{90}$-*co*-NtBAM$_{10}$)

The synthesis of the P(NIPAM$_{90}$-*co*-NtBAM$_{10}$)-NH_2 side chains has been described in a previous publication,[1] in accordance to the synthesis of PNIPAM-NH_2 by Durand et al.[2,3] and P(NIPAM$_{85}$-*co*-NtBAM$_{15}$)-NH_2 by Lencina et al.[4] Briefly, NtBAM (1.25 g, 0.0098 mol) was dissolved in an adequate quantity of DMF. Afterwards, ultrapure water (90 mL) was added progressively in the NtBAM/DMF solution. NIPAM (10 g, 0.0885 mol) was introduced in the mixture and the system was degassed with nitrogen. After that, KPS (2% moles over the monomer concentration) and 2-Mercaptoethylamine hydrochloride (2% and 1% moles over the NtBAM and NIPAM concentration, respectively) were weighted and dissolved separately in 2mL of water and added in the monomers' solution. The synthesis temperature was set at 29 ºC for 24h. Finally, the product copolymer was purified from undesired byproducts and impurities by dialysis membrane (MWCO 12000-14000 Da) against water. The purified copolymer was obtained in a solid state through freeze drying. The molecular characteristics of P(NIPAM$_{90}$-*co*-NtBAM$_{10}$)-NH_2 were

determined in a previous report by proton nuclear magnetic resonance (^{1}H NMR) and acid-base titration while, its cloud point temperature (T_{cp}) was found by turbidimetry (**Table S1**).[1]

Synthesis of the NaALG-g-P(NIPAM$_{90}$-*co*-NtBAM$_{10}$) (NaALG-g) graft copolymer

The grafting of NaALG with the thermo-responsive P(NIPAM$_{90}$-*co*-NtBAM$_{10}$)-NH_2 side chains was achieved using EDC. Firstly, P(NIPAM$_{90}$-*co*-NtBAM$_{10}$)-NH_2 (1.50 g, 0.12 mmol) and NaALG (0.30 g, 1.5 mmol of repeat units) were separately dissolved in 6 mL and 30 mL ultrapure water, respectively, and left to stir at 23 °C until fully dissolved. After complete dissolution, the two polymer solutions were mixed and the coupling agents EDC (0.45 g, 2.3 mmol) and HOBt (0.08 g, 0.60 mmol) were added in the polymers' mixture. The pH of the mixture was set at 6 and the copolymer was left to stir for 3 days at 20 °C. After the 3-day period, the pH of the polymers' solution was increased adding NaOH 1M and the solution was dialyzed by dialysis membrane (MWCO 25000 Da) against water for 5 days. The graft copolymer was obtained in its solid form by freeze-drying. A Bruker Avance III HD Prodigy Ascend TM 600 MHz spectrometer was used to conduct ^{1}H NMR characterization in D_2O and at 20 °C of the NaALG-g copolymer and its precursors NaALG and P(NIPAM$_{90}$-*co*-NtBAM$_{10}$)-NH_2 (**Table S1**).

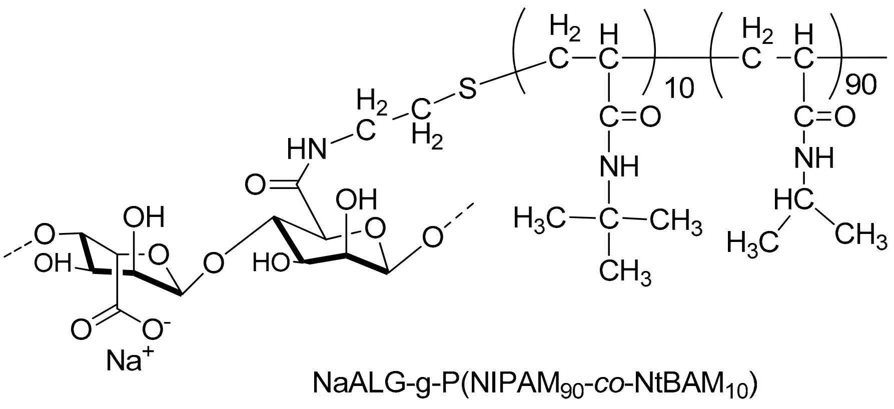

Scheme S1. The structure of the NaALG-g-P(NIPAM$_{90}$-*co*-NtBAM$_{10}$) graft copolymer.

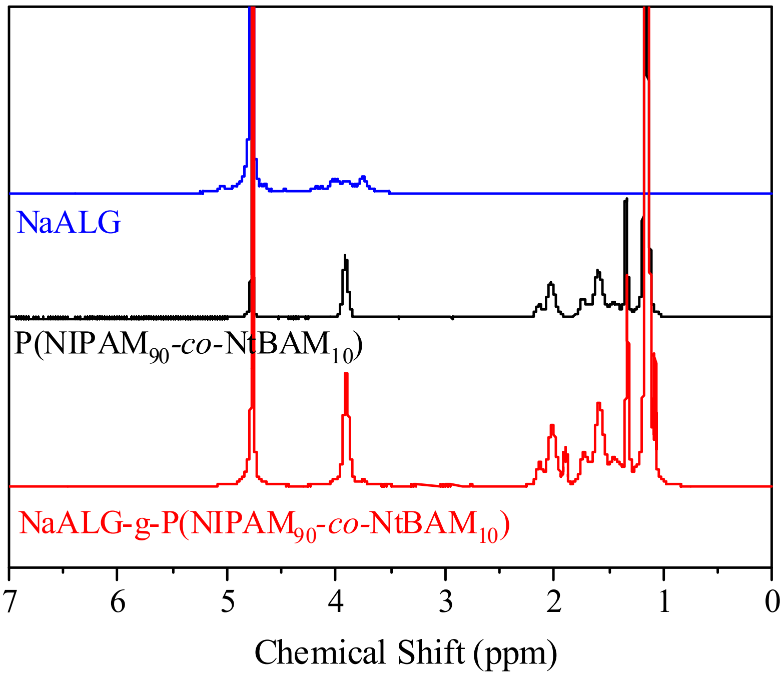

Figure S1. ^{1}H-NMR spectra of NaALG, the side chains P(NIPAM$_{90}$-*co*-NtBAM$_{10}$) and the graft copolymer NaALG-g.

In **Figure S1**, the ^{1}H-NMR spectra of NaALG, P(NIPAM$_{90}$-*co*-NtBAM$_{10}$) and NaALG-g are presented. In all cases, the peak at ~4.8 ppm is attributed to the peak of the deuterated solvent, D_2O. In the ^{1}H NMR spectrum of the polymeric backbone, NaALG, the peaks in the region of 3.5-4.6 ppm are attributed to the four protons of the alginate ring.[5] For the copolymer P(NIPAM$_{90}$-co-NtBAM$_{10}$)-NH_2, one can observe the characteristic peaks of: the proton of the isopropyl group linked to the amide group (N-C-H) at ~3.9 ppm, the methylene and methine protons at the region of 1.3-2.2 ppm, the six methyl protons of the isopropyl group of NIPAM at ~1.1 ppm and the nine methyl protons of the –CH_3 groups of NtBAM at ~1.2 ppm.[6] The composition of the P(NIPAM$_{90}$-co-NtBAM$_{10}$)-NH_2 was calculated from the integration area of the characteristic peaks of the nine methyl protons of the –CH_3 groups of NtBAM at 1.2 ppm and the peak of the proton of the isopropyl group (N-C-H) at ~3.9 ppm.[4,7]

In the NaALG-g spectrum, the characteristic peaks of four alginate protons of NaALG at 3.5–4.3 ppm, overlapped by the N-C-H proton of P(NIPAM-co-NtBAM), the methylene and

methine protons, and the -CH_3 protons of P($NIPAM_{90}$-co-$NtBAM_{10}$) at 1.0-2.2 ppm can be seen. The composition of the graft copolymer was calculated by the comparison of the integrated area at 3.5–4.6 ppm (4H corresponding to NaALG) and the integrated area at 1.0-2.2 (9.3H corresponding to P($NIPAM_{90}$-co-$NtBAM_{10}$). From ^{1}H-NMR it was found that the weight composition of the graft copolymer NaALG-g is NaALG/P($NIPAM_{90}$-co-$NtBAM_{10}$) = 11.5/88.5 (w/w). From this ratio, the grafting density of the copolymer was calculated about 1 side chain P($NIPAM_{90}$-co-$NtBAM_{10}$) per 8 alginate monomer repeating units, which means (taking into account NaALG M_v) that every NaALG chain bears about 86 P($NIPAM_{90}$-co-$NtBAM_{10}$) side chains.

The characterization results are presented in **Table S1**.

Table 1. Molecular characteristics of the polymers.

Polymer	Mv[a] (g/mol)	M_n[b] (g/mol)	M/G ratio[c]	NIPAM/ NtBAM molar ratio[c]	T_{cp} [d] (°C)	weight composition NaALG/grafting chains[c] (w/w)	grafting density: grafting chains/NaALG[c] (mol/mol)
**ALG**	140000	-	1.5	-	-	-	-
**P($NIPAM_{90}$-*co*-$NtBAM_{10}$)-NH_2**	-	12700	-	90/10	25	-	-
**NaALG-g-P($NIPAM_{90}$-co-$NtBAM_{10}$)[e]**	-	-	-	90/10	-	11.5/88.5	86

[a] from intrinsic viscosity; [b] from acid-base titration; [c] from ^{1}H NMR; [d] from turbidimetry, defined at the onset of the optical density abrupt increase; [e] the numbers 90 and 10 in the parenthesis declare the % molar composition of NIPAM/NtBAM monomers in the side chains

Table S2. Characteristics of the SiO_2 particles.

**Particle Size (μm)**	**0.50**
**Surface (m^2/g)**	169.95
**Pore Volume (cm^3/g)**	0.15
**Pore size (nm)**	4.10

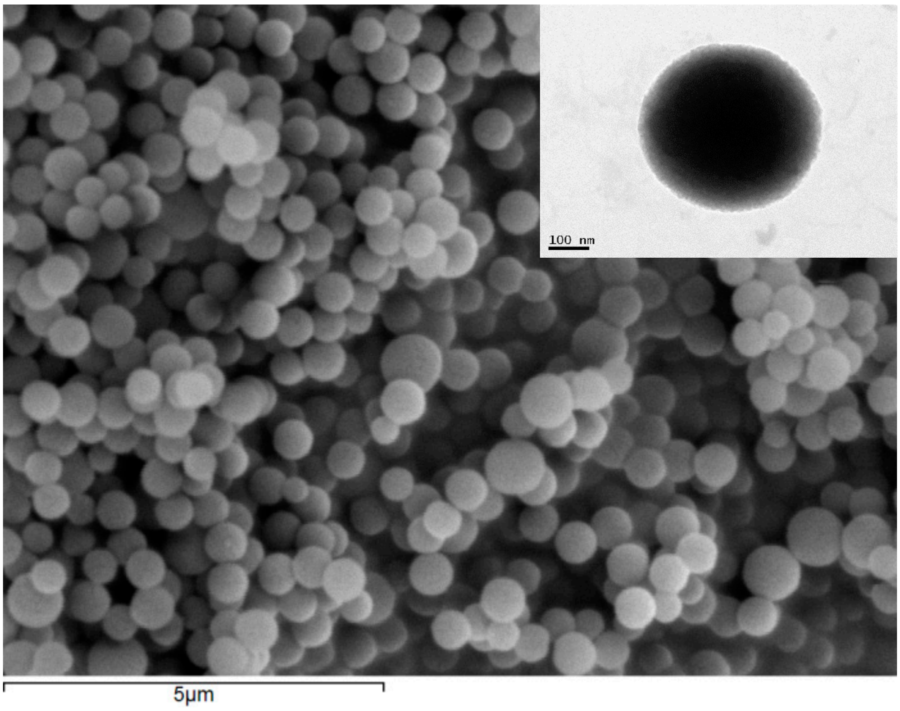

Figure S2. SEM photo of the bare SiO_2 particles. Inset: TEM image of the bare SiO_2 particles.

Table S3. T_{gel} at heating or cooling cycles and hysteresis (ΔT_{gel}) for the copolymer with or without the SiO_2@PAH/NaALG-g hybrid nanoparticles (hNP).

Sample	C_p (wt%)	T_{gel} (ºC) (heating cycle)	T_{gel} (ºC) (cooling cycle)	ΔT_{gel} (ºC)
without nanoparticles: *NaALG-g*	10.0	28.5	27.0	1.5
	7.5	29.1	28.0	1.1
	5.0	31.7	30.1	0.6
with nanoparticles: *hNP@NaALG-g*	5.0	30.7	29.7	1.0

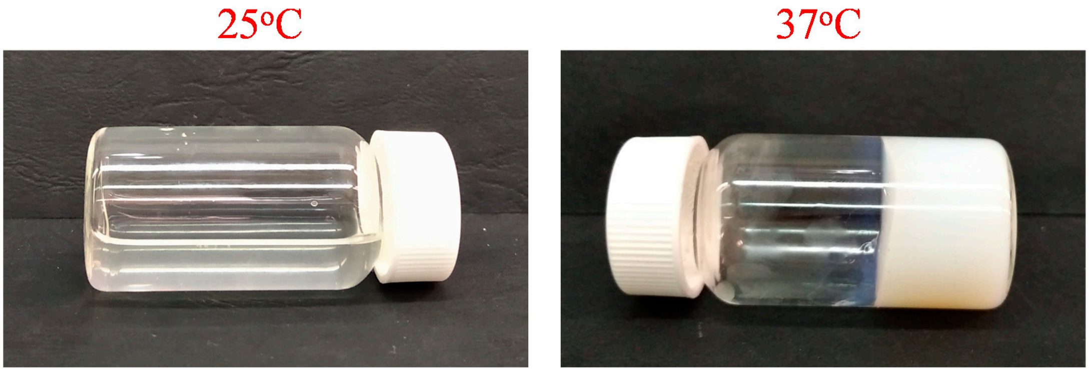

Figure S3. Photos of the NaALG-g: aqueous solution at 25°C and free-standing hydrogel at 37°C.

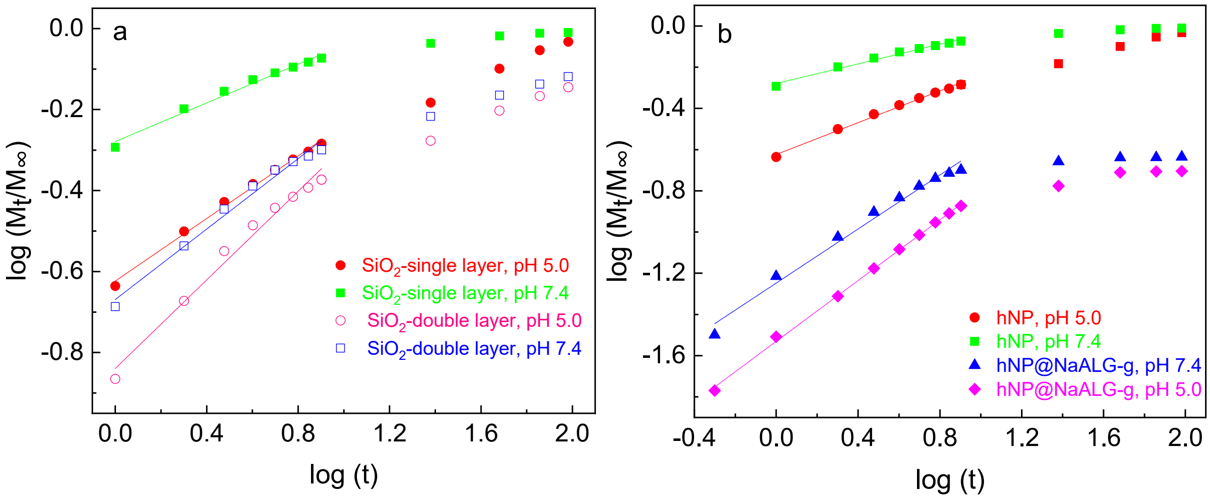

Figure S4. Korsmeyer-Peppas fitting (equation 5) of RB release data: (a) for the SiO_2-single bilayer (hNP) and the SiO_2-double bilayer particles at pH 5.0, pH 7.4 and (b) for the hNP particles and the hNP@NaALG-g composite hydrogel at pH 5.0, pH 7.4, all at T 37 °C. The vertical line denotes 8 hrs.

Table S4. Results of RB release obtained by Korsmeyer-Peppas model fitting (eq. 5).

Sample	Analysis in the time range: 0-8 hours		
	**k_1 (hr^{-n})**	**n**	**R^2**
**pH 5, RBhNP (SiO_2-single bilayer)**	0.238	0.386	0.994
**pH 7.4, RBhNP (SiO_2-single bilayer)**	0.526	0.239	0.981
**pH 5, RB/SiO_2-double bilayer**	0.029	0.547	0.983
**pH 7.4, RB/SiO_2-double bilayer**	0.029	0.435	0.985
**pH 5, RBhNP@NaALG-g**	0.029	0.738	0.999
**pH 7.4, RBhNP@NaALG-g**	0.056	0.656	0.984

References

1 Iatridi, Z.; Saravanou, S.-F.; Tsitsilianis, C. Injectable self-assembling hydrogel from alginate grafted by P(N-isopropylacrylamide-co-N-tert-butylacrylamide) random copolymers. *Carbohydr. Polym.* **2019**, *219*, 344-352.

2 Durand, A.; Hourdet, D. Synthesis and thermoassociative properties in aqueous solution of graft copolymers containing poly(*N*-isopropylacrylamide) side chains. *Polymer* **1999**, *40*, 4941-4951.

3 Durand, A.; Hourdet, D. Thermoassociative graft copolymers based on poly(*N*-isopropylacrylamide): effect of added co-solutes on the rheological behaviour. *Polymer* **2000**, *41*, 545-557.

4 Lencina, M.M.S.; Iatridi, Z.; Villar, M. A.; Tsitsilianis, C. Thermoresponsive hydrogels from alginate-based graft copolymers. *European Polymer J.* **2014**, *61*, 33-44.

5 Guo, H.; de Magalhaes Goncalves, M.; Ducouret, G.; Hourdet, D. Cold and Hot Gelling of Alginate-graft-PNIPAM: A Schizophrenic Behavior Induced by Potassium Salts. *Biomacromolecules* **2018**, *12, 19(2)*, 576-587.

6 Rwei, S.-P.; Chuang, Y.-Y.; Way, T.-F.; Chiang, W.-Y. Thermosensitive copolymer synthesized by controlled living radical polymerization: Phase behavior of diblock copolymers of poly(N-isopropyl acrylamide) families. *J. Appl. Polym. Sci.* **2016**, *133*, 43224.

7 Gibbons, O.; Carroll, W. M.; Aldabbagh, F.; Yamada, B. Nitroxide-mediated controlled statistical copolymerizations of N-isopropylacrylamide with N-tert-butylacrylamide. *J. Polym. Sci., A: Polym. Chem.* **2006**, *44*, 6410–6418.
